# Direct qPCR is a sensitive approach to detect *Mycoplasma* contamination in U937 cell cultures

**DOI:** 10.1186/s13104-019-4763-5

**Published:** 2019-11-01

**Authors:** Zain Baaity, Sven Breunig, Kamil Önder, Ferenc Somogyvári

**Affiliations:** 1 Department of Medical Microbiology and Immunobiology, Faculty of Medicine, University of Szeged, Dóm sq. 10., Szeged, 6720 Hungary; 2 Procomcure Biotech GmbH, Breitwies 1, 5303 Thalgau, Austria

**Keywords:** *Mycoplasma*, qPCR, PCR, Direct, Elimination

## Abstract

**Objective:**

We aim to directly detect *Mycoplasma* DNA in a U937 suspension cell culture without using DNA purification. In order to make *Mycoplasma* contamination monitoring easier, we optimized a commercially available quantitative PCR (qPCR)-based detection kit. We compared the sensitivity of direct qPCR against qPCR with a purified DNA template.

**Results:**

Our findings indicate that qPCR worked optimally with a 6 μl sample volume and a 52 °C annealing-extension temperature. We were able to decrease the annealing-extension step time from 60 to 20 s without any major decrease in reaction sensitivity. The total cycle time of optimized direct qPCR was 65 min. The optimized qPCR protocol was used to detect *Mycoplasma* DNA before and after DNA purification. Our findings indicate that direct qPCR had a higher sensitivity than regular qPCR. Ct levels produced by direct qPCR with 6 μl templates were almost identical to Ct levels produced by regular qPCR with DNA purified from a 60 μl cell culture sample (23.42 vs 23.49 average Ct levels, respectively). The optimized direct qPCR protocol was successfully applied to monitor the elimination of *Mycoplasma* contamination from U937 cell cultures.

## Introduction

*Mycoplasma* is a small cell-wall free prokaryotic bacterium with a remarkable diversity at the species level. Besides causing human respiratory and urogenital tract infections, *Mycoplasma* contamination of cell cultures is a frequent phenomenon. According to the DSMZ-German Collection of Microorganisms and Cell Cultures survey, the prevalence of *Mycoplasma* contamination of cell lines was 28% including *Mycoplasma* species *M. orale*, *M. hyorhinis*, *M. arginini*, *M. fermentans*, *M. hominis* and *Acholeplasma laidlawii* [1]. *Mycoplasma* contamination may be introduced by cross-infection with a *Mycoplasma* positive cell line, laboratory personnel (e.g. *M. orale*) or by contaminated cell-culture reagents such as fetal bovine serum. Indeed, bovine *Mycoplasma* species *M. arginini* and *A. laidlawii* are frequent contaminating agents. *Mycoplasma* contamination is hard to prevent/eradicate since the bacterium is less sensitive to antibiotics commonly applied in cell cultures. Its small size (0.3–1 μm) and non-rigid cell wall makes it also hard to remove by filtration. *Mycoplasma* infection has a pleiotropic effect on cellular physiology including altered metabolism, DNA, RNA and protein synthesis and pro- and anti-inflammatory effects [1–3]. U937 human monocytic cells, the cell-type used in this study, respond to the *Mycoplasma* infection by producing monocyte chemotactic protein-1, matrix metalloproteinase-12 [4] and interleukin-1β [5].

The high probability of introducing novel *Mycoplasma* infections into cell cultures means it is necessary to monitor cell culture ingredients and cell lines for *Mycoplasma* contamination. There are a wide variety of detection methods available including metabolism detection and *Mycoplasma* genome detection by PCR and qPCR. Regular PCR has high sensitivity and specificity, but in the majority of cases requires nucleic acid purification and gel electrophoresis. qPCR eliminates the gel electrophoresis step, but regular qPCR protocols also include nucleic acid purification. DNA purification can be a long and laborious procedure, especially if there are several samples to be purified. Direct PCR and direct qPCR eliminate the purification step, significantly shortening the protocol, but the inhibitory effect of the direct sample can be present. Previously, direct qPCR methods have been successfully applied to monitor *Chlamydia* and herpes simplex virus-2 growth and the antimicrobial effects of various compounds [6–11]. In this study, we want to leave out the DNA purification step and develop a direct qPCR detection method that is suitable to detect *Mycoplasma* contamination within U937 cell cultures.

## Main text

### Materials and methods

#### Cell culture

*Mycoplasma* infected U937 human monocytic cells were grown in an RPMI 1640 medium containing 10% heat-inactivated FBS (Sigma, St. Louis, MO, USA), and 50 μg/mL gentamicin at 37 °C in 5% CO_2_, all within a 25 cm^2^ cell culture flask (Greiner Bio-One Hungary, Mosonmagyaróvár, Hungary).

#### *Mycoplasma* elimination

*Mycoplasma* elimination was performed using *Mycoplasma* Elimination Reagent (Bio-Rad, Hercules, CA, USA). The reagent was added to the RPMI 1640 medium at a 0.5 μg/ml final concentration and the U937 cells were then cultured in this medium for 7 days.

#### DNA extraction and qPCR

DNA was extracted from *Mycoplasma* infected U937 cell supernatants using the Qiagen QIAamp DNA Mini Kit (Qiagen, Hilden, Germany) according to the manufacturer’s instructions. PhoenixDx^®^ Mycoplasma Mix (Procomcure Biotech, Thalgau, Austria) was used in the qPCR experiments. qPCRs with 20 μl final volume were performed using the Bio-Rad CFX Connect qPCR real-time system. A statistical comparison of qPCR cycle threshold (Ct) values was performed with Student’s *t* test, as described previously [12].

### Results

**Fig. 1:**
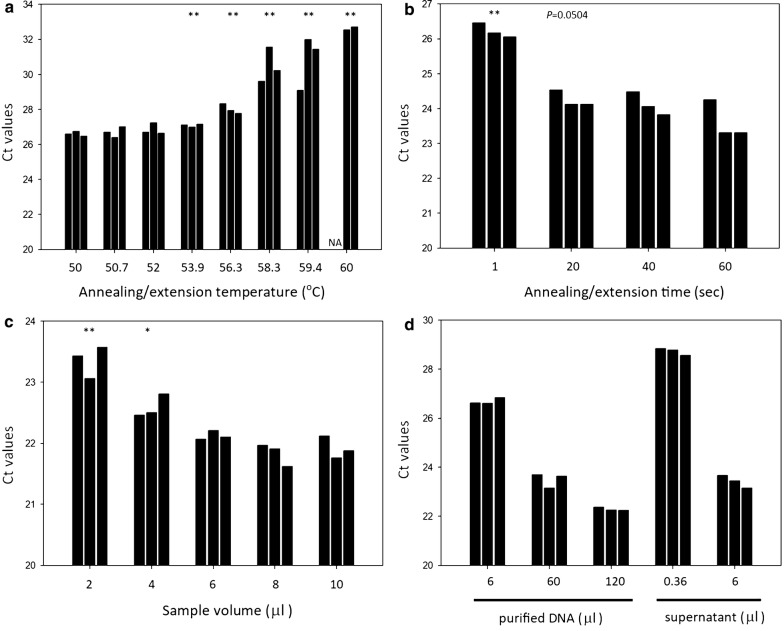
Optimization of *Mycoplasma* genus-specific direct qPCR and comparison of its performance with regular qPCR using purified DNA templates. **a** Effect of the qPCR annealing/extension temperature on the direct qPCR performance. Student’s *t* test was applied to compare the Ct values of samples with various annealing/extension temperatures to those samples with a 50 °C annealing/extension temperature (n = 3). **b** Effect of the annealing/extension time on direct qPCR performance. Student’s t-test was applied to compare the Ct values of the samples with various annealing/extension times to those samples with a 60 s annealing/extension time (n = 4). **c** Effect of sample volume on direct qPCR performance. Student’s t-test was applied to compare the Ct values of various template volume samples with samples having a 10 μl template volume (n = 3). **d** Comparison of direct qPCR performance with regular qPCR using a purified DNA template (n = 3). The DNA was purified from a 6, 60 and 120 μl cell culture supernatant via the QIAamp protocol and eluted in a 100 μl elution buffer. 6 μl of eluted DNA was used in the qPCR procedure. As a comparison, 6 μl of the cell culture supernatant was used in direct qPCR. NA: no amplification was detected. *P < 0.05, **P < 0.01

To achieve optimal sensitivity and the shortest possible reaction time of direct qPCR, we followed a step-wise optimization of the PhoenixDx *Mycoplasma* Mix (Procomcure Biotech, Thalgau, Austria) protocol that was originally designed to amplify purified DNA samples. First, we tested the optimal annealing/extension temperature for detecting unpurified *Mycoplasma* DNA in *Mycoplasma*-infected U937 cell culture supernatants (Fig. 1a). The results indicated that reactions with 50–52 °C annealing/extension temperature produced the lowest Ct values (26.84 ± 0.14–27.06 ± 0.26). We chose the 52 °C annealing/extension temperature for further tests. Next, we tested to see whether reducing the annealing/extension time might influence qPCR performance (Fig. 1b). Our findings showed that the 60 s annealing/extension time provided the lowest Ct values (23.56 ± 0.47), but the 20 and 40 s annealing/extension times led to only slightly higher Ct values (24.20 ± 0.23, 24.11 ± 0.27, respectively), which suggested that reducing the annealing/extension time from 60 to 20 s had a minimal influence on qPCR sensitivity. 20 s annealing/extension time was used for further qPCRs. Next, we tested the effect of sample volume on qPCR performance (Fig. 1c). The Ct levels of samples with 6 μl, 8 μl and 10 μl volumes of supernatants were similar (21.92–22.13 Ct value range), indicating that qPCR sensitivity is influenced by higher *Mycoplasma* DNA content and also by a higher level of qPCR inhibition in the 8 and 10 μl samples. In further experiments, we opted for the 6 μl sample volume. Finally, we compared the performance of direct qPCR and regular qPCR with purified DNA samples (Fig. 1d). The QIAamp DNA purification kit was used to isolate *Mycoplasma* DNA from U937 cell cultures (medium + cells). The elution volume was 100 μl. A comparison of the 6 μl direct sample volume and 6 μl purified sample was not possible as just 6 μl of the 100 μl total elution volume could be used during regular qPCR. Therefore we also decreased the 6 μl direct sample volume by a factor of 6/100 (0.36 μl). In a comparison of these samples we found that the 6 μl purified sample produced lower Ct values (~ 2 cycles) than the 0.36 μl direct sample, suggesting a low level of qPCR inhibition of the supernatant. However, when we compared the Ct levels of samples with 6 μl supernatant to the Ct levels of samples with purified DNAs we noticed that the Ct values produced with 6 μl supernatants were almost identical to those of the purified 60 μl supernatant (23.42 ± 0.26, 23.49 ± 0.30, respectively) indicating an altogether higher sensitivity of the direct qPCR.

**Fig. 2:**
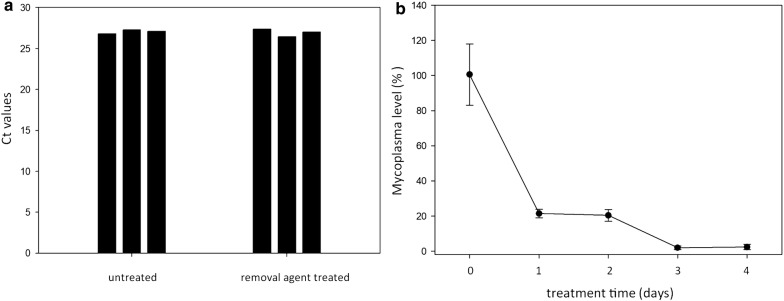
Monitoring *Mycoplasma* elimination by direct qPCR. *Mycoplasma* contaminated U937 cells were treated with Bio-Rad *Mycoplasma* Removal Agent at 0.5 μg/ml concentration. **a** A comparison of qPCR Ct values in the absence and presence of *Mycoplasma* Removal Agent in the medium of contaminated U937 cells. Student’s t-test was applied to compare the Ct values of removal agent containing samples with those of removal agent free samples (n = 3). **b** The first 4 days of treatment monitored by direct qPCR is shown (n = 4 at each time point). The *Mycoplasma* genome concentration on day 0 was defined as 100%

As an application of optimized direct qPCR we monitored *Mycoplasma* elimination from the infected U937 cell culture. Our results showed that the supernatants (n = 4) containing removal agent or free from removal agent both resulted in nearly the same Ct levels (27.04 ± 0.24 and 26.94 ± 0.45, respectively) (Fig. 2a). This indicated that the presence of removal agent did not influence qPCR performance. *Mycoplasma* DNA dropped rapidly (by ~ 80%) after a 24-hour treatment (Fig. 2b). On the fourth day, *Mycoplasma* concentration was 2.3% of the original concentration. By the sixth day of treatment, *Mycoplasma* DNA was no longer detectable (data not shown). Overall, direct qPCR method proved to be a quick and effective method for monitoring the decrease in *Mycoplasma* DNA during the elimination process.

### Discussion

While various methods exist for the detection of *Mycoplasma* contamination [13, 14], probably the most frequently used ones are biochemical detection of *Mycoplasma* metabolism and PCR-based detection of *Mycoplasma* DNA. Though the biochemical detection of mycoplasma ATP generation (Mycoalert (Lonza, Basel, Switzerland)) is a quick protocol, it has certain disadvantages that should be mentioned, including requiring that reagents be reconstituted and brought to 22 °C before each measurement and requiring a luminometer for ATP detection. Aspecificity due to ATP generated by other cells may lead to a high background and eventually false negative measurements. The *Ureaplasma* species which are also a common contaminant in a cell culture [15] cannot be detected by Mycoalert as their own ATP production relies on the hydrolysis of urea [16]. Finally, the sensitivity of biochemical detection has been shown to be lower than that for PCR or qPCR methods [17, 18].

There are a variety of kits on offer based on regular PCR, followed by gel electrophoresis. The major advantage of these kits is the wide availability of regular PCR and electrophoresis equipment. However, decreased specificity compared to probe-based qPCR, the additional electrophoresis step, and the inability to quantitatively monitor the decrease in *Mycoplasma* genome concentration during treatment are clear drawbacks. Intercalation-based (e.g. SYBR Green) qPCR kits such as MycoSEQ *Mycoplasma* Detection Assay (Thermo Fisher, Waltham, MA, USA) eliminate the electrophoresis step and provide quantitative information about *Mycoplasma* genome concentration. The disadvantages of intercalation-based qPCR kits compared to probe-based kits are a lower specificity, lack of internal control and the potential effect of cell culture composition, ionic composition and ionic strength to change the melting temperature of the qPCR product [19–21]. Since this melting temperature is the basis for evaluating specificity in intercalation based qPCRs, changing it can be problematic. Probe-based qPCRs such as PhoenixDx (Procomcure Biotech, Thalgau, Austria), Microsart RESEARCH Mycoplasma (Sartorius, Goettingen, Germany) and qPCR Detection Kit (XpressBio, Frederick, MD, USA) avoid these problems and due the additional requirement of the binding of the probe sequence, these kits provide a higher specificity than regular PCRs and intercalation-based qPCRs.

Noting the advantages of probe-based qPCRs, we optimized the Procomcure PhoenixDx kit to perform a direct qPCR with a *Mycoplasma* infected U937 cell culture. Our results indicates that the optimal temperature was the same as that in the original protocol, so the primer + probe binding was not affected by the presence of the direct template. The fact that the optimal template volume was 6 μl (30% of the total qPCR volume) meant that the direct sample did not have a significant inhibitory effect on the qPCR. A major optimization step that we performed was decreasing the annealing/extension time from 60 s to 20 s, thus saving 40 s in each cycle. Interestingly, this decrease led to only a minor decrease in the sensitivity (~ 0.6 Ct level increase). In addition, decreasing the number of cycles from 50 to 40, reduced the total qPCR time required to 65 min. When we used the optimized qPCR protocol with direct and purified cell culture templates, we found that Ct levels of a 6 μl direct template was almost identical to that of purified DNA from a 60 μl cell culture. The reason for this is mainly due to a dilution of the original DNA content during the elution step at the end of DNA purification. Overall in our case, direct qPCR sensitivity was higher than qPCR with a purified template, with a saving in the cost/time of DNA purification. We monitored the elimination of *Mycoplasma* contamination from the U937 cell culture using the optimized direct qPCR protocol. One of the concerns using pathogen DNA detection is that the non-viable pathogen’s DNA can also be detected and lead to a false positive signal. In our case however, the *Mycoplasma* DNA content dropped to ~ 20% of the original concentration after 1 day of treatment, and though days 1 and 2 contained a similar level of DNA, this decrease continued on day 3. In summary, with direct qPCR we were able to monitor the elimination of *Mycoplasma* over the treatment period.

In conclusion, we optimized a probe-based qPCR to detect *Mycoplasma* contamination in a user-friendly manner. This direct qPCR method does not require a purification step, maintains sensitivity and offers a shorter 65 min protocol.

## Limitations

While we did not observe a major qPCR inhibitory effect of U937 cell culture, it cannot be ruled out that components of other cell cultures may have an inhibitory effect. Most probe based qPCR kits, including the kit used here, contain an internal control (e.g. HEX-labelled probe), therefore the detection of qPCR inhibition (no FAM, no HEX signals) is straightforward. In the case of qPCR inhibition, dilution of the direct sample may be a solution for decreasing/eliminating qPCR inhibition.

## Data Availability

Not applicable.

## Abbreviations

qPCR: quantitative PCR
Ct: cycle threshold
